# Preschoolers of Mothers with Affective and Anxiety Disorders Show Impairments in Cognitive Inhibition During a Chimeric Animal Stroop

**DOI:** 10.9734/INDJ/2013/4193

**Published:** 2013-06-03

**Authors:** Elizabeth A. Calvin, Sharon K. Hunter, Randal G. Ross

**Affiliations:** 1 University of Colorado School of Medicine, Anschutz Medical Campus, Campus Box F546, 13001 E 17th Place, Aurora, Colorado 80045, USA.

**Keywords:** Preschool, executive function, depression, anxiety, attention, stroop

## Abstract

**Aims:**

To determine whether maternal affective and anxiety disorders are associated with cognitive inhibitory deficits in four-year-old children utilizing a chimeric animal stroop task, a childhood adaptation of the traditional stroop task.

**Study Design:**

Blinded Cross-Sectional Study.

**Place and Duration of Study:**

Department of Psychiatry, University of Colorado School of Medicine, data collected from June 2009 to October 2010.

**Methodology:**

Four-year-olds of mothers with (n=29) and without (n=31) a history of affective or anxiety disorders completed a chimeric animal version of the stroop task. Incongruent, neutral, and congruent stimuli were presented over three trial blocks. Mean reaction time and response accuracy were the primary dependent measures.

**Results:**

The increase in the number of incorrect responses to incongruent versus congruent or neutral stimuli was larger for offspring of a mother with a history of an affective or anxiety disorder than without (t=2.4, P=.02); there was no significant main effect of maternal psychiatric illness (F(1, 58)=0.9, P=.34) or a stimulus type by maternal illness (F(1 , 58)=1.1, P=.30) interaction on reaction time.

**Conclusion:**

The association between maternal affective and anxiety disorders and cognitive inhibitory deficit is already identifiable by four years of age.

## 1. INTRODUCTION

Primary prevention and early identification and treatment remain high priority, long-term goals of psychiatric research, and at least two major current trends impact research efforts towards these goals. First, as many symptom clusters in psychiatric disease are common in two or more illnesses and appear related to overlapping etiological factors, there is an increased effort to focus on the symptom cluster, irrespective of disease [1]. Second, as symptoms are often the result of neurodevelopmental processes which begin very early in life, efforts aimed at clarifying when the symptoms develop have increased, with the hope of identifying the developmental window(s) where interventions are likely to have the greatest benefit [2]. In line with these research goals, this study explores whether a specific symptom, cognitive inhibitory dysfunction, is present and identifiable in certain children by four years of age.

In adults and older children, the Stroop Color-Word Test [3] is a standard measure of cognitive inhibitory control. In this task, a participant is presented with the word of a color name written in a different color ink, for example, the word “green” written in red ink. For literate adults, reading is so practiced that when presented with any written word, reading is an easy, seemingly automatic task – a prepotent response. However, when a participant is instructed to not read but to attend to a different portion of the stimulus and provide an alternative response, the task becomes much more difficult. For example, to name the ink of the word’s color, saying “red” instead of reading the word, “green,” the participant must inhibit the automatic or prepotent response and focus elsewhere. When asked to do this, increased response time and errors are noted, illustrating increased difficulty of cognitive inhibition. However, in preschool children under the age of six, decreased literacy and varying degrees of reading fluency alter what would typically be prepotent, decreasing the validity of this task in this population.

Recognition of this developmental difference and the desire to understand the developmental trajectory of cognitive inhibition has spawned multiple preschool adaptations of the stroop task [4-7]. Research utilizing these tasks with typically developing children has yielded similar developmental trends with the results indicating that cognitive inhibitory capacity improves with age in general [4,6,8], but results have been mixed regarding potential cognitive inhibitory deficits while studying preschool-aged populations at risk of attention deficit-hyperactivity disorder [9,10].

The chimeric animal stroop task [5] is one adaptation of the standard stroop task that appears useful for studying inhibitory control in young children. This task takes advantage of a child’s tendency to preferentially identify animals by looking at the head and asks him or her to instead focus on and name the body. By presenting pictures with mismatched animal heads and bodies and asking a child to name the body instead of the head, the inhibitory process is tested in that the participant must give preference to an atypical portion of the stimulus, mirroring the requirements of the color-word stroop [3]. When an image is presented and the head and body are of the same animal, pictures can be easily named. However, when stimuli are incongruent, increased response times and increased errors are seen, again illustrating increased difficulty of cognitive inhibition and reflecting the trends of the color-word stroop in adults [3]. Importantly for developmental research involving preschoolers, this task was found to be most sensitive for children under the age of seven [5].

School-age children (ages seven years and above) of parents with mood, anxiety, and psychotic disorders have increased rates of cognitive inhibitory deficits and general attention problems [11-20]. The mechanisms mediating this relationship are not completely understood; however, both prenatal maternal physiology and maternal caregiving may contribute in a dynamic way to this observed correlation. [21-23]. However, utilizing previous test batteries, inhibitory dysfunction was not prominent in preschoolers and manifested for many children suddenly at school age [24]. Currently, it is unclear if the lack of symptomatology in preschool studies is secondary to a delay in manifestation of symptoms until school age, or if previous tests lacked sensitivity that more recent childhood testing adaptations may be capable of detecting.

The primary objective of this study was to determine if differences could be detected in four-year-olds at risk of cognitive inhibitory deficits secondary to maternal affective or anxiety disorders utilizing the chimeric animal stroop [5], a preschool adaptation of the traditional color-word stroop task [3]. To the authors’ knowledge, this is the first comparative examination of preschool age children of mothers with and without psychiatric disorders utilizing this task.

## 2. METHODOLOGY

### 2.1 Participants

The Colorado Infant Development Project is a longitudinal study focusing on the relationship between parental mental illness and early cognitive development. Infants and their parents are recruited through the Colorado State Department of Vital Statistics from the Denver metropolitan region. Structured maternal psychiatric assessments of parents are completed within a few months of the child’s birth. From this initial sample, children were re-recruited around four years of age to undergo neurocognitive testing. Recruitment was limited to those families for whom English was their primary language. This report covers children born between June 2005 and October 2006; 60 of 95 (63%) children recruited at infancy completed the neurocognitive test battery at four years of age. A total of sixty four year olds of mothers with (N=29) and without (N=31) a history of affective or anxiety disorders were examined. No children were taking psychoactive medications at the time of testing.

### 2.2 Study Design

#### 2.2.1 Maternal psychiatric illness

Mothers were interviewed while the child was a young infant (generally under three months of age) utilizing a structured diagnostic instrument, the Structured Clinical Interview for DSM-IV [25]. An experienced clinician (MSW or MD) completed all interviews. Diagnoses were best estimate diagnoses. A positive history of an affective or anxiety disorder was defined as an Axis I depressive (Major Depressive Disorder or Depression NOS; n=21) or anxiety disorder (PTSD, OCD, GAD, or Panic Disorder; n=15). Three mothers also had a comorbid history of mania (n=2) or psychosis (n=1). Analyses were completed both including and excluding mothers with a comorbid history of bipolar or psychotic illness and produced similar results. Analyses including these mothers are reported here. Focal anxiety disorders, such as specific and social phobias, were not considered as a positive history.

#### 2.2.2 Neurocognitive testing

The neurocognitive test battery was administered across two sessions. On the initial testing visit, children were administered a Wechsler Preschool and Primary Scale of Intelligence [26]. This visit occurred at 4 years of age (mean +S.D of 48.9+ 0.7 months; range 47.9 - 51.2 months) and was followed by a second visit for completion of other neurocognitive tasks at an average of 14.9 days (SD = 11.6 days, range 0-49 days) after the initial visit. In addition to the chimeric animal stroop, the experimental protocol initially included the kiddie continuous performance test (KCPT) [27] and the flexible item selection task (FIST) [28]. Many children were unable to complete the KCPT task and performance on the FIST task was at ceiling with all children performing well. Part way through data collection, the FIST was replaced with the dimensional change card sort (DCCS) task [29], the number of children who had completed this task was too low to provide sufficient power to justify analysis. As such, analysis was limited to the chimeric animal stroop task.

#### 2.2.3 Chimeric animal stroop

As a pre-test, all children were shown laminated paper pictures of, and asked to name, each of the four utilized matching stimuli (a sheep, a cow, a duck, and a pig). Their responses in this pre-test were utilized to determine later correct and incorrect responses. For example, some children used “lamb” instead of “sheep,” and one child utilized animal sounds instead of names (“baa,” “moo,” “quack” and “oink”). A portable laptop computer utilizing software provided by Ingram Wright [5] presented stimuli for the task. On a separate computer monitor placed in front of the child, stimuli measuring 7cm by 9 cm were presented in one of two orientations, i.e., head facing to the left or right of the screen.

**Fig. 1.**
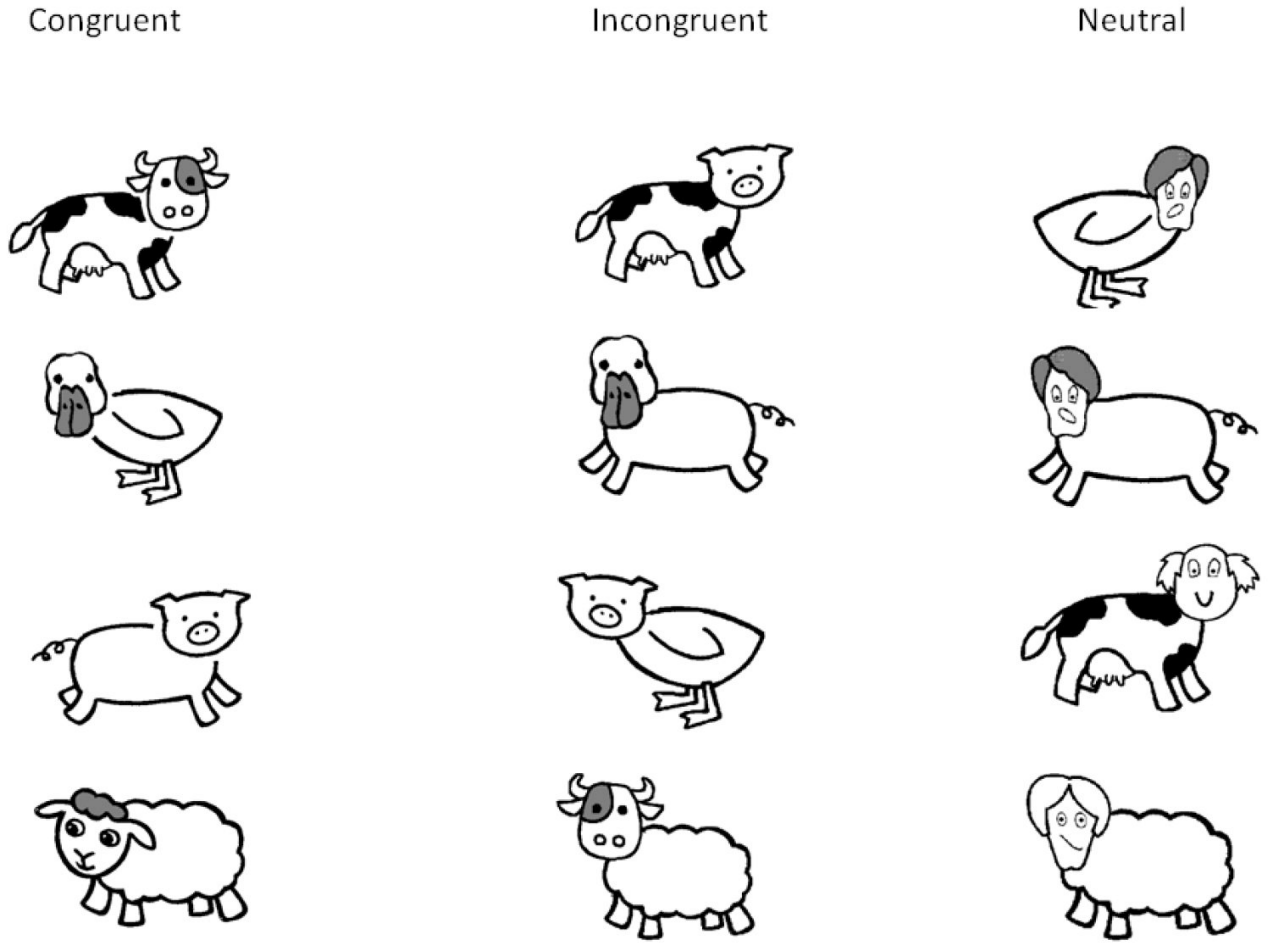
Examples of stimuli used by type

In order to orient participants to the computer monitor and encourage vocal responses, a series of 6 pictures of common objects were presented, i.e., a car, train, bus, race car, and boat. Following this familiarization phase, a chimeric animal with a cat head and horse body was presented on the monitor. The examiner explained that the child would be shown pictures of “funny animals where the head and body don’t match,” and the child was instructed to “name just the body” of each animal. The testing session consisted of three blocks of stimuli: the first and third blocks each contained 24 pseudo-randomly presented incongruent (mismatched animal head and body) and neutral (cartoon face with animal body) stimuli. The second block contained 24 matching (congruent) stimuli. Fig. 1 provides examples of presented images.

Each trial began with a fixation point in the center of the screen for 500 ms and was followed by presentation of a chimeric animal image for three seconds or until 500 ms after the examiner utilized a key press marking the child’s first animal syllabic response. Following a one second inter-stimulus interval, a new trial began with presentation of the fixation point. Videotapes were reviewed, and each child’s responses were categorized as correct, incorrect, or no-response by an examiner blinded to each child’s maternal history.

### 2.3 Setting

For stroop testing, children were videotaped while being tested in a quiet research room in the presence of their accompanying parent. Maternal diagnostic testing and Wechsler Preschool and Primary Scale of Intelligence testing occurred in the same facility prior to stroop testing as noted above.

### 2.4 Variables

Reaction time was recorded by the examiner’s key press with each child’s first initial syllabic response. Responses were later viewed by an examiner blinded to each child’s group and were scored as “incorrect” if the child responded with a name other than that of the body. Each child’s initial syllabic response was given preference, for example, “sh…duck” for a sheep-duck chimera with a duck body was counted as an incorrect response. Trials where the child failed to respond at all or responded outside of the three-second window were counted as no-response (errors of omission) and were not included in further analyses. The analysis focused on errors of commission, defining performance as the percentage of correct responses: number of correct responses / (number of correct + number of incorrect responses).

### 2.5 Data sources/Measurement

Reaction time (via examiner’s key press) was recorded utilizing software provided by Ingram Wright [5]. An independent blinded examiner coded responses as correct, incorrect, or no-response as above.

### 2.6 Bias

All examiners (reviewing data and preforming testing) were blinded as to each child’s allocated group (maternal affective or anxiety disorder or no maternal psychiatric history).

### 2.7 Quantitative Variables

Maternal affective or anxiety disorder vs no maternal psychiatric history were determined as noted in study design above. Upon review, it was noted that many children had a large variation in participation across blocks with some children appearing to initially not understand the task and others losing interest and ceasing participation. To account for this variability, the block where each child had the best participation rate (least percentage of no response answers) was utilized for analysis. Blocks where a child did not respond to at least 80% of stimuli (three blocks in two children without a maternal psychiatric illness and one with) were eliminated from further analyses. Reaction time was calculated as an average across each grouping.

### 2.8 Statistical Methods

SPSS [30] was utilized to complete repeated measure ANOVAs with maternal psychiatric history (yes versus no for Axis I disorders) as a between-subjects factor and stimulus type (congruent, neutral, and incongruent) as a within-subjects factor. While gestational age at birth was not statistically different between groups, this is potentially an important factor. Thus, analyses were run both with and without gestational age as a covariate, and the results were similar. Results without gestational age as a covariate are reported here.

## 3. RESULTS AND DISCUSSION

Twenty-nine (48%) of the participants had a mother who had a positive history of an affective or anxiety disorder prior to or during the child’s early infancy. No statistically significant differences were found between infants with and without a positive maternal history in gender, gestational age, birth-weight, race / ethnicity, social environment, intelligence scores, maternal age, history of pregnancy complications, parental education, paternal history of Axis I diagnosis, or maternal smoking exposure (Table 1), and all mothers denied a history of substance or alcohol abuse during pregnancy.

### 3.1 Reaction Times

**Fig. 2.**
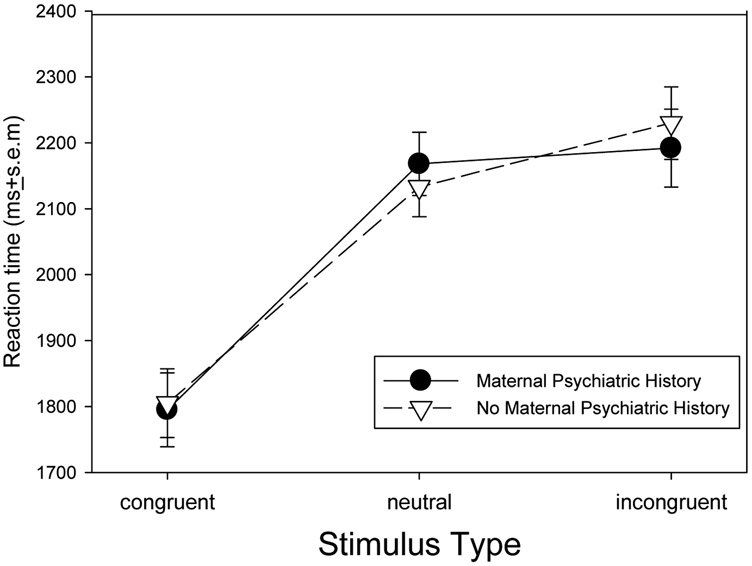
Mean reaction times (ms) ± standard error of the mean (s.e.m.) by stimulus type. Reaction times for congruent stimuli are faster than for either neutral or incongruent stimuli (*P* < .001), but not signficantly different between neutral and incongruent stimuli. There was no significant difference between offspring with (n=27) and without (n=31) maternal psychiatric history or a stimulus type by psychiatric history interaction.

Reaction times were analyzed using a 2 x 3 (Group [no maternal psychiatric history, positive maternal psychiatric history] x Stimulus Type [congruent, neutral, incongruent]) repeated measures ANOVA. Fig. 2 demonstrates a strong main effect for trial type, *F*(2, 116) = 26.3, *P*<.001, where mean reaction times for congruent stimuli were faster than for neutral or incongruent stimuli, *P*<.001; however there was no significant difference in mean reaction times between the neutral and incongruent stimuli, *P*=.82. There was also no significant main effect for maternal psychiatric history, *F*(1, 58)=0.9, *P*=.34, or maternal psychiatric history by trial type interaction, *F*(1 , 58)=1.1, *P*=.30.

### 3.2 Response Accuracy

Responses were analyzed using a 2 x 3 (Group [no maternal psychiatric history, positive maternal psychiatric history] x Stimulus Type [congruent, neutral, incongruent]) repeated measures ANOVA. The percentage of correct responses was defined as the number of correct responses / (number of correct + number of incorrect responses).

**Fig. 3.**
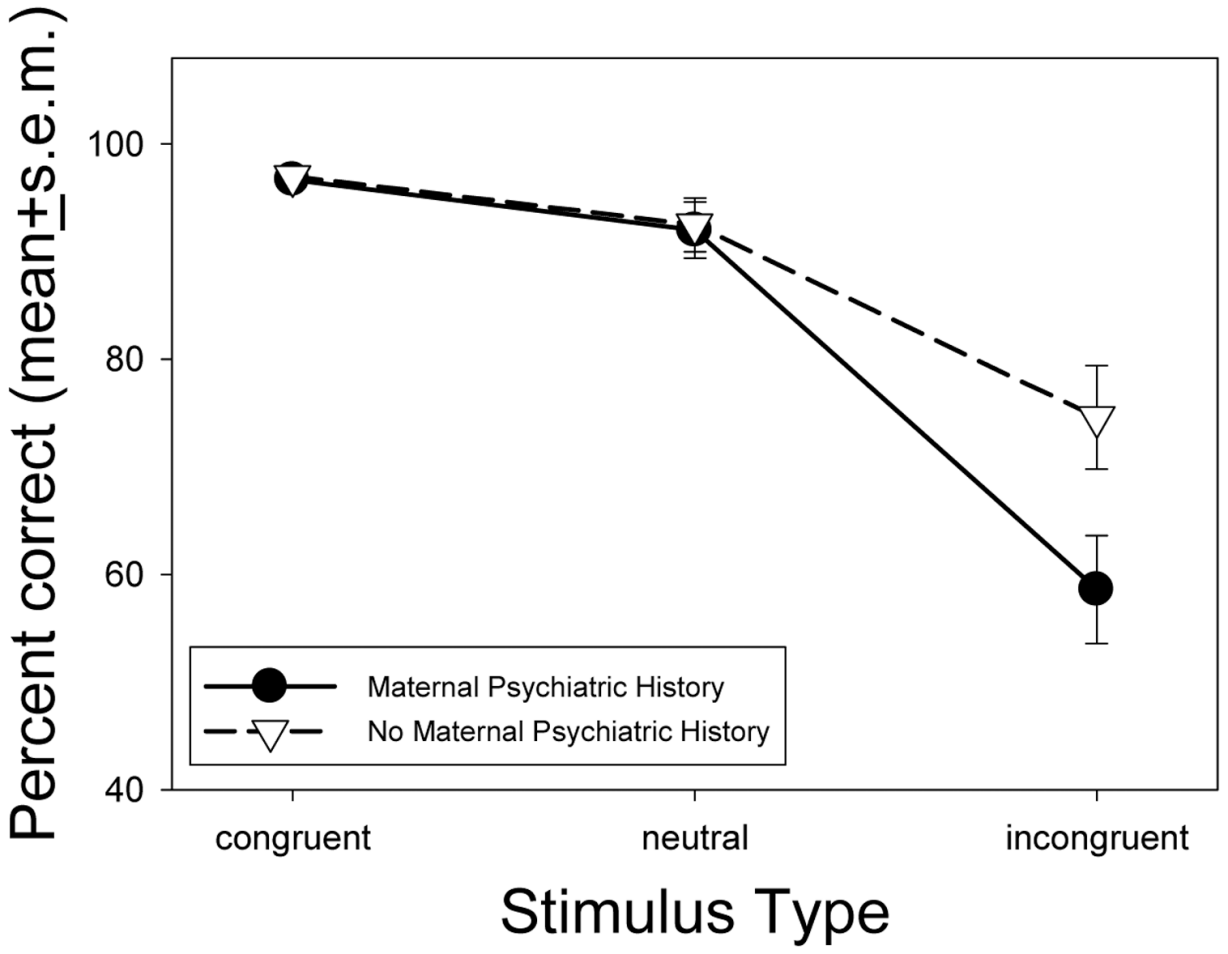
Percentage of correct responses + standard error of the mean (s.e.m.) by stimulus type. Percentages did not significantly differ by maternal psychiatric history for congruent or neutral stimuli (*P*’s =.79 - .93); however offspring with a mother with a positive psychiatric history (n=29) had lower correct response rates to incongruent stimuli than did offspring with a mother with a negative psychiatric history (n=31; P=.02)

There was a significant main effect of stimulus type, *F*(2, 116) = 83.4; *P* < .001. The number of correct responses was lower for the incongruent condition than either the congruent, *P*<.001, or neutral conditions, *P*<.001, and there were also fewer correct responses in the neutral than the congruent condition, *P*=.02. There was a trend for a main effect of group, *F*(1, 58) = 3.4, *P*=. 07, and a significant stimulus-type by group interaction, *F*(1, 58)=5.85; *P*=.02. There was no effect of group on percentage of correct responses when the stimuli were congruent, *t*=0.0, *P*=1.0, or neutral, *t*=0.1, *P*=.94. While both groups had a lower correct response percentage in response to incongruent stimuli, the effect was larger for the group with a positive maternal psychiatric history such that the children of mothers with positive psychiatric histories performed significantly poorer than the negative maternal psychiatric history group, *t* = 2.4, *P*=.02, (Fig. 3).

We did not have enough power in this data set to examine affective and anxious maternal psychiatric diagnoses individually. However, a preliminary analysis demonstrated that there was a significant trial type by maternal psychiatric history interaction wherein, compared to offspring of a mother with a negative psychiatric history, significantly poorer performance was observed both in children of a mother with a history of an anxiety disorder (Generalized Anxiety Disorder, Obsessive Compulsive Disorder, Post-Traumatic Stress Disorder, and/or Panic Disorder (*n*=15)), *F* (2, 88) =4.8, *P*=.03, and in children of a mother with a depressive disorder (Major Depressive Disorder and/or Depression Not Otherwise Specified (*n*=21)), *F*(2, 100)=4.4, *P*=.04.

The purpose of this study was to assess the relationship between a positive maternal psychiatric history of affective or anxiety disorders and a measure of cognitive inhibitory dysfunction in preschool-aged offspring. Our results indicate that specific inhibitory deficits are identifiable by four years of age. As expected, when the animal head and body matched (congruent trials), mean reaction times were the fastest and most children approached 100% accuracy. When the head of the stimulus was a neutral face-like drawing (neutral trials), there was a slowing of reaction time with a slight decrease in accuracy. During incongruent trials, when the animal head and animal body were mismatched, accuracy dropped off, with a greater drop for offspring of mothers with a positive psychiatric history. This indicates more difficulty for children of mothers with an affective or anxiety disorder to inhibit their prepotent response, and this difficulty is profound enough to be seen at a time in which all children have a developmentally limited capacity to do so.

In this sample, there was no difference between groups in exposing their fetuses to toxins (substance abuse and smoking); however, mothers with a history of psychiatric illness are generally at higher risk for tobacco and other substance use. Subtle differences between groups in maternal substance use cannot be entirely ruled out as contributors to offspring differences. In addition, maternal mental illness and child offspring inhibitory deficits may also share genetic risk factors. It is also possible that specifically anxiety or depression may have a larger effect on cognitive inhibition in offspring. Though we did not have enough power in our study to examine specific diagnoses, preliminary analysis did show an effect of both depressive and anxiety disorders wherein children of mothers with these diagnoses, compared to children whose mothers did not have a psychiatric disorder, illustrated a greater deficit in cognitive inhibitory control. More research with a larger population is needed to determine if deficits seen are disorder specific, genetically predisposed, or if the effect on children is secondary to a more general factor, such as stress, which may change the mother’s in-utero environment and/or contribute to difficulty with early attachment to offspring. It is also unknown whether there is a critical window, minimum duration, or severity of symptoms for maternal illness to effect the offspring.

The mechanism by which maternal illness is related to child inhibitory performance is unknown. Child psychopathology, whether because of shared genetic risk factors or because of maternal illness driven changes in the child’s environment, is one possibility. In this study, children were not themselves screened for psychopathology, and thus this possibility cannot be investigated here. Future efforts should include assessment of child psychopathology.

Performance on the chimeric animal stroop task was as expected; children responded most rapidly and with high accuracy to congruent trials, took longer to respond to neutral trials with a slight decrease in accuracy, and then had accuracy significantly diminish with incongruent trials. This pattern is indicative of the difficulty and higher cognitive function associated with inhibiting an increasingly prepotent response. However, as children could choose not to respond, and no response answers were not analyzed, it is difficult to know what their reaction times would have been if a response was required. For example, it is possible that some children might have needed longer than three seconds to process incongruent stimuli, and certainly upon video review, responses given after the three second cut-off were seen. If this were the case, differences between groups may be larger than identified. Additionally, some children may also simply give up and not respond to more difficult tasks, potentially skewing the data to misrepresent children with less inhibitory deficit or those trials that are simply easier to inhibit.

As the previously published chimeric animal stroop was created to assess the development of cognitive inhibition with age, it is also possible that further modification of the paradigm will produce more robust detection of cognitive inhibitory deficit when comparing similarly aged children. By changing to a forced-response model and providing additional time for children to respond, further deficits and differences between categories may become apparent. Additionally, from a developmental perspective, inhibitory capacity has been shown to improve with age in the chimeric animal stroop [5,32], and it would be interesting to see if the observed inhibitory deficits remain constant, diminish or increase over time in the identified cohort and how their development compares to that of controls.

Regardless of potential maternal and child cognitive etiologies, early identification of children with cognitive inhibitory deficit has important lasting implications. For example, adult psychotic, depressive, anxious, and bipolar symptomatology is often preceded by attentional deficits as a child [33,34], and difficulties in inhibitory capacity in childhood have also been seen in child and adolescent psychiatric illnesses including depression, attention deficit / hyperactivity disorder, bipolar disorder, generalized anxiety disorder, adjustment disorder, conduct and oppositional defiant disorder, and post-traumatic stress disorder [35-42]. Identification of at risk groups at an earlier age may provide the opportunity for enhanced monitoring and earlier diagnosis and treatment. Even if children identified do not eventually develop psychiatric illness, inhibitory deficits have also been associated with decreased social competence, non-cooperative behavior, and conduct problems [39,43,44], as well as educational difficulties that negatively impact lives. Some attempts to increase inhibitory control and cognitive flexibility in children have been successful (with greatest gains being seen in those with the poorest skills at baseline) [45]. With earlier identification, particularly at a time of high synaptic plasticity and prior to school initiation, the opportunity becomes available to intervene when even small modifications may have the potential to induce long-term change.

## 4. CONCLUSION

The association between maternal affective and anxiety disorders and cognitive inhibitory deficit is already identifiable by four years of age. More research is needed to determine the mechanism explaining the relationship between maternal affective and anxiety disorders and offspring inhibitory deficits. Further adaptation of the chimeric animal stroop may produce even more robust detection of cognitive inhibitory deficit. As cognitive inhibitory deficit is a risk factor for later psychiatric illness and is associated with decreased social functioning, early identification provides the opportunity to intervene when small modifications may induce great long-term change.

## Figures and Tables

**Table 1.** Demographic representation of participants

Characteristic	No Maternal Psychiatric Illness (n=31)	Maternal Psychiatric Illness (n=29)	t or X^2^ Value
**Male Gender (%)**	Males: 13 (41.9)	Males: 14 (48.2)	*X^2^* (1, *n* = 60) = 0.05, *P* = .82
**Age at Stroop testing (months)**	*M* = 48.7 *SD* = 0.6	*M* = 49.0 *SD* = 1.0	*t* (58) = 1.5, *P* = .22
**Pregnancy Complications (%)**	Yes: 8 (25.8)	Yes: 10 (34.5)	*X^2^* (1, *n* = 60) = 0.2, *P* = .65
**Gestational Age at Birth (days)**	M = 276 SD = 10	*M* = 270 *SD* = 19	*t* (58) = 2.1, *P* = .15
**Birth Weight (grams)**	*M* = 3189.8 *SD* = 513.2	*M* = 3201.7 *SD* = 669.3	*t* (58) = 0.0, *P* = .94
**Race / Ethnicity (%)**	Caucasian / Non-Hispanic: 27 (87.1) Other: 4 (12.9)	Caucasian / Non-Hispanic: 22 (75.9) Other: 7 (24.1)	*X^2^* (1, *n* = 60) = 0.62, *P* = .43
**Parental Social Environment (%)**	Lives with Biological Parents: 31 (100)	Lives with Biological Parents: 28 (96.6)	*X^2^* (1, *n* = 60) = 0.0, *P* = 1.0
**Maternal Age at Child’s Birth (years)**	*M* = 32 *SD* = 5	*M* = 33 *SD* = 5	*t* (58) = 0.6, *P* = .44
**Maternal Education (years)**	*M* = 16 *SD* = 2	*M* = 17 *SD* = 2	*t* (58) = 0.1, *P* = .73
**Paternal Education (years)**	*M* = 16 *SD* = 2	*M* = 16 *SD* = 3	*t* (58) = 0.4, *P* = .56
Maternal socioeconomic status*	*M* = 53 *SD* = 22	*M* = 55 *SD* = 23	*t* (58) = 0.1, *P* = .74
Paternal socioeconomic status*	*M* = 61 *SD* = 18	*M* = 61 *SD* = 25	*t*(58) = 0.0, *P* = .96
**Maternal Smoking Exposure (%)**	Yes: 2 (6.5)	Yes: 4 (13.8)	*X^2^* (1, *n*=60) = 0.27, *P* = .60
**WPPSI Intelligence Score (*SD*)**	Verbal Composite *M* = 111 (15)	Verbal Composite *M* = 108 (9)	*t* (58) = 1.0, *P* = .31
	Performance Composite *M* = 107 (12)	Performance Composite *M* = 105 (12)	*t* (58) = 0.7, *P* = .41
	Processing Speed Composite *M* = 97 (9)	Processing Speed Composite *M* = 94 (10)	*t*(58) = 1.8, *P* = .19
	Full Composite *M* = 109 (10)	Full Composite *M* = 106 (11)	*t*(58) = 1.5, *P* = .22
**Maternal Axis I Diagnosis (%)**	No: 31 (100) Yes: 0 (0)	No: 0 (0) Yes: 29 (100) Major Depression: 19 Depression NOS: 2 PTSD: 5 Obsessive-Compulsive: 3 Generalized Anxiety: 8 Panic Disorder: 5	
**Paternal Axis I Diagnosis (%)**	Yes: 6 (19.4) No: 22 (71.0) Unknown: 3 (9.7)	Yes: 8 (27.6) No: 18 (62.1) Unknown: 3 (10.3)	*X^2^* (1, *n* = 60) = 0.62; *P* = .73

* Socioeconomic status is based on is based on The Socioeconomic Index of Occupations [31]. 503 occupations are included and are scored in a potential range of 0-100. Managerial and professional occupations generally have scores above 60; technical, sales, and administrative support occupations generally score between 35 and 60; service, agricultural, and labor occupations generally have scores below 35. Scores are based on the highest occupation value achieved across an individual’s life.

## References

[1] Insel T, Cuthbert B, Garvey M, Heinssen R, Pine DS, Quinn K, Research domain criteria (RDoC): toward a new classification framework for research on mental disorders. Am J Psychiatry. 2010;167:748–751. United States. 20595427 10.1176/appi.ajp.2010.09091379

[2] National Advisory Mental Health Council. Transformative Neurodevelopmental Research in Mental Illness. National Institute of Mental Health; 2008.

[3] Stroop JR. Studies of interference in serial verbal reactions. Journal of Experimental Psychology. 1935;18(6):643–662.

[4] O'Brien-Quinn S, Quinn EP. The Stroop Interference Effect in Young Children: A Developmentally Appropriate Approach. Individual Dif Res. 2005;3(3):183–187.

[5] Wright I, Waterman M, Prescott H, Murdoch-Eaton D. A new Stroop-like measure of inhibitory function development: typical developmental trends. J Child Psychol Psychiatry. 2003;44(4):561–575. 12751848 10.1111/1469-7610.00145

[6] Prevor MB, Diamond A. Color-object interference in young children: A Stroop effect in children 3(1/2)-6(1/2) years old. Cognitive Development. 2005;20(2):256–278. 18079980 10.1016/j.cogdev.2005.04.001 PMC2134842

[7] Gerstadt CL, Hong YJ, Diamond A. The relationship between cognition and action: Performance of children 3 1/2 - 7 years old on a Stroop-like day-night test. Cognition. 1994;53:129–153. 7805351 10.1016/0010-0277(94)90068-x

[8] Montgomery DE, Koeltzow TE. A review of the day-night task: The Stroop paradigm and interference control in young children. Developmental Review. 2010;30:308–330.

[9] Berwid OG, Curko Kera EA, Marks DJ, Santra A, Bender HA, Halperin JM. Sustained attention and response inhibition in young children at risk for Attention Deficit / Hyperactivity Disorder. J Child Psychol Psychiatry. 2005;46(11):1219–1229. 16238669 10.1111/j.1469-7610.2005.00417.x

[10] Thorell LB, Wahlstedt C. Executive functioning deficits in relation to symptoms of ADHD and/or ODD in preschool children. Inf Child Develop. 2006;15:503–518.

[11] Ozan E, Deveci E, Oral M, Karahan U, Oral E, AydIn N, Neurocognitive functioning in a group of offspring genetically at high-risk for schizophrenia in Eastern Turkey. Brain Research Bulletin. 2010;82(3-4):218–223. 20435102 10.1016/j.brainresbull.2010.04.013

[12] Gotlib IH, Traill SK, Montoya RL, Joormann J, Chang K. Attention and memory biases in the offspring of parents with bipolar disorder: indications from a pilot study. J Child Psychol Psychiatry. 2005;46(1):84–93. 15660646 10.1111/j.1469-7610.2004.00333.x

[13] Souzzia JM, Motta RW. The Relationship Between Combat Exposure and the Transfer of Trauma-like Symptoms to Offspring of Veterans. Traumatol. 2004;10(17):17–37.

[14] Moradi AR, Neshat-Doost HT, Teghavi R, Yule W, Dalgleish T. Performance of children of adults with PTSD on the stroop color-naming task: a preliminary study. J Traum Stress. 1999;12(4):663–671. 10.1023/A:1024721218869 10646184

[15] Clavarino AM, Mamun AA, O'Callaghan M, Aird R, Bor W, O'Callaghan F, Maternal Anxiety and Attention Problems in Children at 5 and 14 Years. Journal of Attention Disorders. 2010;13(6):658–667. 19805622 10.1177/1087054709347203

[16] Hay DF, Pawlby S, Sharp D, Asten P, Mills A, Kumar R. Intellectual Problems Shown by 11-year-old Children Whose Mothers Had Postnatal Depression. J Child Psychol Psychiatry. 2001;42(7):871–889. 11693583 10.1111/1469-7610.00784

[17] Tully EC, Iacono WG, McGue M. An Adoption Study of Parental Depression as an Enviornmental Liability for Adolescent Depression and Childhood Disruptive Disorders. 2008;165(9):1148–1154. 10.1176/appi.ajp.2008.07091438 PMC2573034 18558644

[18] Talge NM, Neal C. Antenatal maternal stress and long-term effects on child neurodevelopment: how and why? J Child Psychol Psychiatry. 2007;48(3-4):245–261. Blackwell Publishing Ltd. 17355398 10.1111/j.1469-7610.2006.01714.x PMC11016282

[19] Van den Bergh BRH, Marcoen A. High antenatal maternal anxiety Is related to ADHD symptoms, externalizing problems, and anxiety in 8- and 9-year-olds. Child Dev. 2004;75(4):1085–1097. Blackwell Publishing. 15260866 10.1111/j.1467-8624.2004.00727.x

[20] Erlenmeyer-Kimling L, Cornblatt BA, Rock D, Roberts S, Bell M, West A. The New York High-Risk project: anhedonia, attentional deviance, and psychopathology. Schizo Bull. 1993;19(1):141–153. 10.1093/schbul/19.1.141 8451608

[21] Hunter SK, Mendoza JH, D'Anna KL, Zerbe GO, McCarthy L, Hoffman C, Antidepressants may mitigate the effects of prenatal maternal anxiety on infant auditory sensory gating. Am J Psychiatry;2012. 10.1176/appi.ajp.2012.11091365 PMC3640273 22581104

[22] Bagner DM, Pettit JW, Lewinsohn PM, Seeley JR. Effect of maternal depression on child behavior: a sensitive period? J Am Acad Child Adolesc Psychiatry. 2010;49:699–707. 20610139 10.1016/j.jaac.2010.03.012 PMC2901251

[23] Love JM, Kisker EE, Ross C, Raikes H, Constantine J, Boller K, The effectiveness of Early Head Start for 3-year-old children and their parents: Lessons for policy and programs. Dev Psychol. 2005;41(6):885–901. US, American Psychological Association. 16351335 10.1037/0012-1649.41.6.88

[24] Lavigne JV, Arend R, Rosenbaum D, Binns HJ, Christoffel KK, Gibbons RD. Psychiatric disorders with onset in the preschool years: I. Stability of diagnoses. J Am Academy Child Adolesc Psychiatry. 1998;37:1246–1254. 10.1097/00004583-199812000-00007 9847496

[25] First MB, Spitzer RL, Gibbon M, Wingerson DK. Structured clinical interview for DSM-IV Axis I Disorders - Patient Edition; New York, New York State Psychiatric Institute; 1997.

[26] Weschler D Weschler Preschool and Primary Scale of Intelligence, 3rd Edition, San Antonio, TX, USA, Psychological Corporation; 2002.

[27] Conners CK, Staff M. Conners Kiddie Continuous Performance Task (K-CPT): Computer Program for Windows Technical Guide and Software Manual. Toronto, ON, Canada, Multi-Health Systems, Inc.; 2001.

[28] Jacques S, Zelazo PD. The flexible item selection task (FIST): A measure of executive function in preschoolers. Dev Neuropsychol. 2001;20:573–591. 12002094 10.1207/S15326942DN2003_2

[29] Zelazo PD. The Dimensional Change Card Sort (DCCS): a method of assessing executive function in children. Nat Protocols. 2006;1(1):297–301. Nature Publishing Group. 17406248 10.1038/nprot.2006.46

[30] PASW Statistics. Chicaco, IL, USA, IBM. 2010;18.

[31] Nakao K, Treas J. The 1989 socioeconomic index of occupations: construction from the 1989 occupational prestige scores. General Social Survey Methodological Report No. 74, Chicago, University of Chicago, National Research Center; 1992.

[32] Nichelli F, Scala G, Vago C, Riva D, Bulgheroni S. Age-Related Trends in Stroop and Conflicting Motor Response Task Findings. Child Neuropsychol. 2005;11:431–443. 16306018 10.1080/09297040590951569

[33] Erlenmeyer-Kimling L, Rock D, Roberts SA, Janal M, Kestenbaum C, Cornblatt B Attention, memory, and motor skills as childhood predictors of schizophrenia-related psychoses: the New York High-Risk Project. Am J Psychiatry. 2000;157(9):1416–1422. 10964857 10.1176/appi.ajp.157.9.1416

[34] Davidson M, Reichenberg A, Rabinowitz J, Weiser M, Kaplan Z, Mark M. Behavioral and intellectual markers for schizophrenia in apparently healthy male adolescents. Am J Psychiatry. 1999;156(9):1328–1335. 10484941 10.1176/ajp.156.9.1328

[35] Cataldo M, Nobile M, Lorusso M, Battaglia M, Molteni M. Impulsivity in depressed children and adolescents: A comparison between behavioral and neuropsychological data. Psychiatry Res. 2005;136:123–133. 16125790 10.1016/j.psychres.2004.12.012

[36] Qian Y, Shuai L, Cao Q, Chan RC, Wang Y. Do executive function deficits differentiate between children with attention deficit hyperactivity disorder (ADHD) and ADHD comorbid with oppositional defiant disorder? A cross-cultural study using performance-based tests and the behavior rating inventory of executive function. Clin Neuropsychol. 2010;24(5):793–810. 20582856 10.1080/13854041003749342

[37] Kilic BG, Sener S, Kockar AI, Karakas S. Multicomponent attention deficits in attention deficit hyperactivity disorder. Psychiatry Clin Neurosci. 2007;61:142–148. 17362431 10.1111/j.1440-1819.2007.01629.x

[38] Doyle AE, Wilens TE, Kwon A, Seidman L, Faraone SV, Fried R Neuropsychological Functioning in Youth with Bipolar Disorder. Biol Psychiatry. 2005;58:540–548. 16199011 10.1016/j.biopsych.2005.07.019

[39] Homack S, Riccio CA. A meta-analysis of the sensitivity and specificity of the Stroop Color and Word Test with children. Arch Clin Neuropsychol. 2004;19(6):725–743. 15288327 10.1016/j.acn.2003.09.003

[40] Taghavi MR, Dalgleish T, Moradi AR, Neshat-Doost HT, Yule W. Selective processing of negative emotional information in children and adolescents with Generalized Anxiety Disorder. 2003;42(Pt 3):221–230. 10.1348/01446650360703348 14565889

[41] Golden ZL, Golden CJ. Patterns of Performance on the Stroop Color and Word Test in Children with Learning, Attentional, and Psychiatric Disabilities. Psychol Schols. 2002;39(5):489–495.

[42] Moradi AR, Taghavi MR, Neshat Doost HT, Yule W, Dalgleish T. Performance of children and adolescents with PTSD on the Stroop colour-naming task. Psychol Med. 1999;29(2):415–419. 10218932 10.1017/s0033291798008009

[43] Ciairano S, Visu-Petra L, Settanni M. Executive inhibitory control and cooperative behavior during early school years: a follow-up study. J Abnorm Child Psychol. 2007;35(3):335–345. 17226093 10.1007/s10802-006-9094-z

[44] Rhoades B, Greenberg M, Domitrovich C. The contribution of inhibitory control to preschoolers' social-emotional competence. J Appl Dev Psychol. 2009;30:310–320.

[45] Diamond A, Lee K. Interventions shown to aid executive function development in children 4 to 12 years old. Science. 2011;333(6045):959–964. 21852486 10.1126/science.1204529 PMC3159917

